# Supplementary material for: Multi‐dataset identification of innovative feature genes and molecular mechanisms in keratoconus
Source: J Cell Mol Med. 2024 Sep 19;28(18):e70079. doi: 10.1111/jcmm.70079 (PMC11412914; doi:10.1111/jcmm.70079)
Supplement: Supplementary file 2 — Material S2. [file JCMM-28-e70079-s002.zip › 7.GSEA analysis/2.ATOH7/KEGG_ADHERENS_JUNCTION.html]

Details for gene set KEGG\_ADHERENS\_JUNCTION[GSEA]

|  || Dataset | ATOH7.group.cls#h\_versus\_l |
| Phenotype | group.cls#h\_versus\_l |
| Upregulated in class | h |
| GeneSet | KEGG\_ADHERENS\_JUNCTION |
| Enrichment Score (ES) | 0.38983417 |
| Normalized Enrichment Score (NES) | 1.4655293 |
| Nominal p-value | 0.104743086 |
| FDR q-value | 0.2638112 |
| FWER p-Value | 0.827 |
Table: GSEA Results Summary

  

Fig 1: Enrichment plot: KEGG\_ADHERENS\_JUNCTION      
 Profile of the Running ES Score & Positions of GeneSet Members on the Rank Ordered List

  

| SYMBOL | TITLE | RANK IN GENE LIST | RANK METRIC SCORE | RUNNING ES | CORE ENRICHMENT || 1 | FYN | na | 470 | 0.560 | 0.0645 | Yes |
| 2 | FGFR1 | na | 573 | 0.527 | 0.1351 | Yes |
| 3 | LEF1 | na | 735 | 0.492 | 0.1992 | Yes |
| 4 | PTPRB | na | 1455 | 0.390 | 0.2329 | Yes |
| 5 | SNAI2 | na | 2981 | 0.282 | 0.2282 | Yes |
| 6 | ACTN2 | na | 2998 | 0.281 | 0.2669 | Yes |
| 7 | TCF7 | na | 3059 | 0.277 | 0.3039 | Yes |
| 8 | SRC | na | 4033 | 0.235 | 0.3087 | Yes |
| 9 | SSX2IP | na | 4355 | 0.223 | 0.3305 | Yes |
| 10 | ACTN1 | na | 4698 | 0.211 | 0.3502 | Yes |
| 11 | PTPRM | na | 5367 | 0.189 | 0.3573 | Yes |
| 12 | ACTN3 | na | 5743 | 0.178 | 0.3714 | Yes |
| 13 | MET | na | 6191 | 0.165 | 0.3815 | Yes |
| 14 | TCF7L1 | na | 6644 | 0.153 | 0.3898 | Yes |
| 15 | WASF3 | na | 7566 | 0.129 | 0.3813 | No |
| 16 | CTNNA3 | na | 8333 | 0.112 | 0.3747 | No |
| 17 | WAS | na | 9234 | 0.095 | 0.3620 | No |
| 18 | EP300 | na | 10138 | 0.079 | 0.3470 | No |
| 19 | PTPN6 | na | 10373 | 0.076 | 0.3508 | No |
| 20 | TGFBR1 | na | 11693 | 0.057 | 0.3206 | No |
| 21 | VCL | na | 11955 | 0.053 | 0.3205 | No |
| 22 | MAP3K7 | na | 12201 | 0.050 | 0.3204 | No |
| 23 | EGFR | na | 12485 | 0.046 | 0.3187 | No |
| 24 | CSNK2B | na | 13092 | 0.039 | 0.3067 | No |
| 25 | CSNK2A2 | na | 13289 | 0.037 | 0.3063 | No |
| 26 | FER | na | 13306 | 0.037 | 0.3110 | No |
| 27 | RAC2 | na | 13916 | 0.031 | 0.2978 | No |
| 28 | YES1 | na | 14068 | 0.030 | 0.2976 | No |
| 29 | MAPK1 | na | 14536 | 0.025 | 0.2877 | No |
| 30 | WASF1 | na | 15259 | 0.019 | 0.2695 | No |
| 31 | RHOA | na | 15382 | 0.018 | 0.2685 | No |
| 32 | RAC1 | na | 16331 | 0.010 | 0.2425 | No |
| 33 | SORBS1 | na | 16752 | 0.007 | 0.2314 | No |
| 34 | MAPK3 | na | 16821 | 0.006 | 0.2303 | No |
| 35 | ACTG1 | na | 16994 | 0.005 | 0.2259 | No |
| 36 | WASF2 | na | 17673 | -0.001 | 0.2065 | No |
| 37 | CDC42 | na | 17874 | -0.003 | 0.2012 | No |
| 38 | SMAD3 | na | 18063 | -0.005 | 0.1964 | No |
| 39 | WASL | na | 18186 | -0.006 | 0.1937 | No |
| 40 | CTNNB1 | na | 18214 | -0.006 | 0.1937 | No |
| 41 | PTPN1 | na | 18450 | -0.008 | 0.1880 | No |
| 42 | INSR | na | 18814 | -0.011 | 0.1791 | No |
| 43 | CTNNA1 | na | 18820 | -0.011 | 0.1806 | No |
| 44 | NLK | na | 19287 | -0.015 | 0.1693 | No |
| 45 | CDH1 | na | 19700 | -0.019 | 0.1600 | No |
| 46 | TGFBR2 | na | 20164 | -0.023 | 0.1499 | No |
| 47 | PARD3 | na | 20314 | -0.025 | 0.1491 | No |
| 48 | IQGAP1 | na | 20581 | -0.027 | 0.1453 | No |
| 49 | ACP1 | na | 21349 | -0.036 | 0.1281 | No |
| 50 | PTPRF | na | 21634 | -0.039 | 0.1253 | No |
| 51 | ACTN4 | na | 21725 | -0.040 | 0.1283 | No |
| 52 | SMAD4 | na | 22118 | -0.044 | 0.1231 | No |
| 53 | TCF7L2 | na | 22134 | -0.044 | 0.1289 | No |
| 54 | ACTB | na | 22412 | -0.047 | 0.1275 | No |
| 55 | LMO7 | na | 22573 | -0.049 | 0.1298 | No |
| 56 | CTNNA2 | na | 22741 | -0.051 | 0.1321 | No |
| 57 | CTNND1 | na | 23789 | -0.067 | 0.1112 | No |
| 58 | PTPRJ | na | 24059 | -0.071 | 0.1133 | No |
| 59 | ERBB2 | na | 24092 | -0.071 | 0.1223 | No |
| 60 | CSNK2A1 | na | 24568 | -0.078 | 0.1194 | No |
| 61 | TJP1 | na | 24758 | -0.081 | 0.1254 | No |
| 62 | CREBBP | na | 24831 | -0.083 | 0.1348 | No |
| 63 | SMAD2 | na | 25145 | -0.088 | 0.1380 | No |
| 64 | BAIAP2 | na | 26905 | -0.123 | 0.1045 | No |
| 65 | FARP2 | na | 27610 | -0.141 | 0.1038 | No |
| 66 | IGF1R | na | 27632 | -0.142 | 0.1230 | No |
| 67 | SNAI1 | na | 31444 | -0.266 | 0.0501 | No |
| 68 | RAC3 | na | 32406 | -0.320 | 0.0670 | No |
Table: GSEA details [plain text format]

  

Fig 2: KEGG\_ADHERENS\_JUNCTION      
 Blue-Pink O' Gram in the Space of the Analyzed GeneSet

  

Fig 3: KEGG\_ADHERENS\_JUNCTION: Random ES distribution      
 Gene set null distribution of ES for **KEGG\_ADHERENS\_JUNCTION**
